# Two commodity queueing inventory system with random common lifetime, two demand classes and pool of customers

**DOI:** 10.1016/j.heliyon.2023.e21478

**Published:** 2023-10-28

**Authors:** S. Dissa, P.V. Ushakumari

**Affiliations:** Department of Mathematics, Amrita School of Physical Sciences, Amrita Vishwa Vidyapeetham, Amritapuri Campus, 690525, Kerala, India

**Keywords:** Inventory, Two commodity, Common lifetime, Matrix geometric method

## Abstract

This paper analyzes a two-commodity perishable queueing inventory system with random common lifetimes commodity-wise, positive service time, and individual ordering policy. We have considered two types of customers: priority customers and non-priority customers. There is a buffer of finite capacity for the priority customers and a pool with infinite capacity made available for the non-priority customers. The arrival of these customers is occurred by two independent Poisson processes. The lifetimes of each of the commodities and service times follow independent exponential distributions. The matrix geometric approach is used to analyze the model's steady-state behavior and we computed various system performance measures. This article examines the effects of the common lifetime parameters on the measures of performance, such as average cycle time, the average number of reorders due to the realization of the common lifetime of commodities, etc. Also, a sensitivity analysis of the system parameters on the performance measures is carried out. Further, a cost function is constructed based on the performance measures, and its convexity is examined numerically.

## 1 Introduction

This paper covers the study of a two-commodity perishable inventory system with two different categories of items, two types of customers, a random common lifetime (CLT) commodity-wise, positive service time, individual ordering policy, and instantaneous replenishment (zero lead time) for commodities. Inventory control is one of the most predominant business processes leaned on by businesses, as they reconcile their undertakings coordinated with purchase, sales, and strategic marketing activities. One of the factors that affect the prevailing inventory management is the group of items stocked. The management of inventory systems with multi-commodity items poses arduous challenges compared to their single-item counterpart inventory systems, ensued by the collection of inventoried items and correlated activities.

Diligent management of perishable products is imperative and calls for companies to be run judiciously. These obsolete products impose time and cost premiums to change or even worse, to be disposed of. Such major problems are rampant in the food or healthcare industry where products perish easily on the manufacturing line, in storage, or during delivery. This precipitates intricacies in the distribution of inventory as per customer demands. Besides, there are variations in demand, lifetime, or customer behavior. Smart inventory management of perishable products obviates wastage, with an augmented likelihood of distribution of goods fulfilling customer demands.

In perishable inventory systems, items with finite lifetimes can be divided into two categories. In the first category, each of the items has an independent lifetime, and in the second category, items that survive a random common lifetime in inventory. In the second category, when the random common lifetime realizes all items of that commodity perish together. There are numerous applications of inventory models in this category. Food items such as fresh fruits, vegetables, meats, pharmaceuticals produced in batches, photographic materials, and even electronic things like memory chips are examples of perishable inventory items that require batch replacement when their shelf life runs out. Another example of a common lifetime inventory situation is cloud data storage. Nowadays cloud storage has become an essential part of any business operation. On cloud storage, the data can be accessed only for the period it is purchased, for example, on over-the-top (OTT) platforms, where the content they provide has a limited span in terms of relevancy and viewer desire. The main objective of this research is to analyze the effect of the random common lifetime parameters on the measures of performance, such as average cycle time and average number of reorders due to the realization of the common lifetime of commodities of a two-commodity perishable inventory system.

The rest of the paper is organized as follows: Section 2 gives literature review. Model formation and analysis are given in section 3. Section 4 provides performance measures such as the expected number of customers, expected reorder rates, average sojourn time of type-1 and type-2 customers, and cycle time for two types of commodities. In section 5, cost analysis is conducted and numerical illustrations are provided. Section 6 gives conclusion.

## 2 Literature review

Stochastic inventory system with single and multi-commodity literature is very extensive. In this section, we provide some of the important research work carried out in two commodity stochastic inventory systems and perishable inventory systems. A two commodity inventory system in which each demand is for one unit of commodity-1, one unit of commodity-2, or one unit of each of the commodities with prefixed probabilities was studied by [1]. Inventory problems of two commodities with Markov shift in demands were investigated by [2] and [3]. In those models, a new demand for an item at each demand epoch follows a discrete-time Markov chain. Investigations of two commodity inventory systems involving joint and individual replenishments of items were carried out by [4], [5], and [6]. A two commodity problem with a joint replenishment policy and different order quantities was studied by [7]. Also [8], [9] and [10] analyzed two commodity inventory systems with substitutable items. An inventory system with two commodities and renewal demands was examined by [11]. The same authors analyzed the system with retrial demand in [12]. Two commodity queueing inventory systems with positive service times were studied in papers [13] and [14]. A perishable inventory system with a modified (S−1,S) policy and arbitrary processing times, was investigated by [15]. Later the same authors in [16], studied a lost sales (s,S) type perishable inventory system under continuous review, with varying demands on ordering quantity and renewal demands. A perishable inventory system under the (s,Q) policy and the Markovian arrival process of demands along with a finite buffer was discussed by [17]. In that paper, the authors assumed a single server who begins service of items only after N customers have accumulated in the system. Exceptionally good reviews of the modeling expeditions of single commodity perishable inventory systems were provided in [18], [19], [20], [21], and [22]. Also for an extensive literature survey on inventory models with positive service time, one can refer to [23].

Some of the notable research work carried out in two commodity perishable inventory literature are as follows. A two-commodity perishable inventory system at a service facility, where each item in inventory followed an exponentially distributed lifetime, was examined in [24]. A two-commodity perishable inventory system with three categories of customers and exponentially distributed lifetimes for each of the commodities was analyzed by the authors [25] and a multi-criteria inventory management system for perishable and substitutable products was considered by authors [26]. In all those papers, the authors discussed inventory models with either fixed life time of items or items that have a random lifetime that fails independently of other items. As we discussed in the beginning, it is essential to study inventory models with random common lifetimes due to their applications. However, very few studies have been done on such kinds of models. The first reported work on inventory systems in which items have a random common lifetime was attributed to the study referenced as [27]. They analyzed the inventory system at discrete points in time. Later [28] studied a perishable inventory system with a random common lifetime of items and Markovian demands in continuous time. Queuing inventory systems with a random common lifetime of items were analyzed by [29] and [30]. All the mentioned work with random common lifetime of items were on single commodity inventory systems. As far as our knowledge goes, no such work has been reported in the literature on multi-commodity inventory systems. Present-day inventory management is very complex due to the multitude of items stocked and this complexity increases when items are perishable and have a random common lifetime in inventory. To avoid the wastage of materials and getting perfect customer service, the study of such systems is essential. We have examined such a scenario in this work.

## 3 Model formation and analysis

Consider a two-commodity perishable queueing inventory system at a service facility. Demands coming to the system occur according to two independent Poisson processes with rates $\lambda_{1}$ and $\lambda_{2}$, calling them type-1 and type-2 customers, respectively. It is assumed that type-1 customers are non-priority customers, type-2 customers are priority customers, and type-1 customers demand commodity-1 alone, and type-2 customers demand commodity-2 alone. The maximum inventory level for commodity *i* at the beginning of a cycle is $S_{i},i=1,2$, and all items are assumed to be fresh at the start of the replenishment epoch. It presumes that inventoried items have a random common lifetime (CLT), which indicates that all items expired together on realization of this time. We assume that the random common lifetime of commodity-*i* is exponentially distributed with rate $\gamma_{i},i=1,2$, and is independent of each other. The inventory policy is $\left(s_{i},S_{i}\right)$ type for commodity-i,i=1,2, instantaneous replenishment of items, and individual ordering for commodities. Whenever the inventory level reaches $s_{i}$, a reorder for $S_{i}$ items is placed for commodity-i,i=1,2. This is slightly different from the conventional (s,S) type systems. Any items remaining in inventory at the time of replenishment will be removed to accommodate the new batch of items. For priority customers, there is a buffer size of finite capacity, say M(>0). At the time of the arrival of a priority customer, the buffer is full, and that customer is assumed to be lost. Non-priority customers can either join a pool of infinite capacity with probability *p* or exit the service area with probability (1−p) when arrival finds a busy server. Using the assumption of a pool for non-priority customers, the server will get the status of those customers, even though there are no buffer size allocations for non-priority customers. Service times are independent, exponentially distributed random variables, with rates $\mu_{1}$ and $\mu_{2}$ for commodities 1 and 2, respectively. On execution of the service, the idle server selects any customers from the buffer on a first-come, first-served (FCFS) basis. When there are no customers in the buffer, customers from the pool will be served on a FCFS basis. It is assumed that perishability is applicable only to the inventoried items and not to customers in queue and also assumed non-preemptive priority for service.

**Note:**

• In our model, inventory reaches its maximum level of $S_{i}$ after replenishment. This can happen in two ways: either due to the realization of CLT or because the inventory level reaches $s_{i}$ due to demand. To distinguish the attainment of replenishment due to the realization of CLT, we use the temporary notation $S_{i}^{⁎}$, which is equal to $S_{i}$.

### 3.1 The mathematical model

For the mathematical formulation of the model, we define the following: At time t,

$N_{1}(t)$: the number of customers in the pool including the one in service, if any.

$N_{2}(t)$: the number of customers in the buffer including the one in service, if any.

$I_{1}(t)$: the on hand inventory level of commodity-1

$I_{2}(t)$: the on hand inventory level of commodity-2 and

S(t): the state of the server at time *t*, where

$S(t)=\left\{ \begin{array}{c} 0\;,\;\text{if the server idle at time t}\\ 1\;,\;\text{if the server busy with a type-1 customer at time t}\\ 2\;,\;\text{if the server busy with a type-2 customer at time t} \end{array}\right.\;$

Let $Z(t)=(N_{1}(t),N_{2}(t),I_{1}(t),I_{2}(t),S(t))$. Then {Z(t);t≥0} forms a continuous time Markov chain over the state space $E=\{(0,0,i_{1},i_{2},0);i_{1}=s_{1},...,S_{1},S_{1}^{⁎};i_{2}=s_{2},...,S_{2},S_{2}^{⁎}\}⋃\{(n_{1},n_{2},i_{1},i_{2},1);i_{1}=s_{1},...,S_{1},S_{1}^{⁎};i_{2}=s_{2},...,S_{2},S_{2}^{⁎};n_{1}\geq 0;0\leq n_{2}\leq M;M\geq 1\}⋃\{(n_{1},n_{2},i_{1},i_{2},2);i_{1}=s_{1},...,S_{1},S_{1}^{⁎};i_{2}=s_{2},...,S_{2},S_{2}^{⁎};n_{1}\geq 0;0\leq n_{2}\leq M;M\geq 1\}$ where $S_{i}^{⁎},i=1,2$ denote a temporary state which shows the inventory level just after replenishment due to the attainment of CLT. This is same as $S_{i},i=1,2$. However to distinguish the beginning of the next cycle due to realization of common lifetime, we use it as a purely temporary notation. Consider the number of customers in the pool as the level. By the above assumptions and notations, {Z(t),t≥0} forms a level independent Quasi-Birth and Death (LIQBD) process over E. The level $\left\{(0,0,i_{1},i_{2},0);i_{1}=s_{1},...,S_{1},S_{1}^{⁎};i_{2}=s_{2},...,S_{2},S_{2}^{⁎}\right\}$ is of dimension $\left(S_{1}-s_{1}+2)(S_{2}-s_{2}+2\right)$ which signifies to the case when the server is idle and there is no waiting customer in the queue and the on hand inventory level of commodity-1 is $i_{1}$ and the on hand inventory level of commodity-2 is $i_{2}$. The level $\left\{(n_{1},n_{2},i_{1},i_{2},1);i_{1}=s_{1},...,S_{1},S_{1}^{⁎};i_{2}=s_{2},...,S_{2},S_{2}^{⁎};n_{1}\geq 0;0\leq n_{2}\leq M;M\geq 1\right\}$ of dimension $\left(S_{1}-s_{1}+2)(S_{2}-s_{2}+2)(M+1\right)$ signifies to case where a non priority customer is with the server and there are $n_{2}$ priority customers in the buffer and $n_{1}$ non-priority customers in the pool and also the inventory levels for priority and non-priority customers are $i_{2}$ and $i_{1}$ respectively. Also the level $\left\{(n_{1},n_{2},i_{1},i_{2},2);i_{2}=s_{1},...,S_{1},S_{1}^{⁎};i_{2}=s_{2},...,S_{2},S_{2}^{⁎};n_{1}\geq 0;0\leq n_{2}\leq M;M\geq 1\right\}$ is of dimension $\left(S_{1}-s_{1}+2)(S_{2}-s_{2}+2)(M+1\right)$ signifies to the case that a priority customer is being served and there are $n_{2}$ priority customers in the buffer and $n_{1}$ non-priority customers waiting in the pool and also the inventory level for priority and non-priority customers are $i_{2}$ and $i_{1}$ respectively.

The infinitesimal generator matrix of the Markov chain of the system is of the form

$$Q=\left[\begin{array}{cccccc} B_{00} & B_{01} & & & & \\ B_{10} & A_{2} & A_{1}\\ & A_{3} & A_{2} & A_{1}\\ & & A_{3} & A_{2} & A_{1}\\ & & & \ddots & \ddots & \ddots \end{array}\right]$$

Let us denote $\;\;d_{1}=(S_{1}+2-s_{1})\;(S_{2}+2-s_{2})$

$d_{2}=(S_{1}+2-s_{1})\;(S_{2}+2-s_{2})(M+1)\;;\;\;d_{3}=2d_{2}\;;\;\;\;a=(S_{1}+2-s_{1})\;\;;\;b=(S_{2}+2-s_{2})$

$I_{d_{1}}$ denote an identity matrix of order $d_{1}$. The symbol ⨂ represents the Kronecker product of matrices. **I** denote an identity matrix of appropriate order. **e** is a column vector of one's with appropriate order. The notation **0** represents a zero matrix of appropriate order. With these notations, the sub matrices of **Q** are as follows:

$B_{01}={\left[\begin{array}{c} 0\\ A_{1} \end{array}\right]}_{(d_{3}+d_{1})\times d_{3}};\;\;B_{00}={\left[\begin{array}{cc} C_{00} & C_{01}\\ C_{02} & A_{2}^{\prime } \end{array}\right]}_{\left(d_{3}+d_{1})\times (d_{3}+d_{1}\right)};\;\;B_{10}={\left[\begin{array}{cc} 0 & A_{3} \end{array}\right]}_{d_{3}\times (d_{3}+d_{1})}$

$A_{1}=I_{2(M+1)}⨂E;\;\;E=(\lambda_{1}p)I_{d_{1}};\;\;C_{01}={\left[\begin{array}{cccccccccc} A_{01} & 0 & 0 & ... & 0 & A_{02} & 0 & 0 & ... & 0 \end{array}\right]}_{d_{1}\times d_{3}}$

$C_{1}=\mu_{1}I_{d_{1}};\;\;C_{2}=\mu_{2}I_{d_{1}};\;\;C_{02}={\left[\begin{array}{cccccccccc} C_{1} & 0 & 0 & ... & 0 & C_{2} & 0 & 0 & ... & 0 \end{array}\right]}_{d_{1}\times d_{3}}^{T};J_{1}={\left[\begin{array}{cc} 0 & E_{1}\\ I & 0\\ E_{2} & 0 \end{array}\right]}_{b\times b}$

$E_{1}=[1\;0]\;;\;\;E_{2}={\left[0\;0\;...\;0\;1\right]}_{1\times (b-2)}\;;\;\;J=\lambda_{2}J_{1}$

$A_{01}=J_{1}⨂\lambda_{1}I_{b}\;\;\;\;;\;A_{02}=I_{a}⨂J\;\;\;\;;\;H=J_{1}⨂\mu_{1}I_{b}\;\;\;\;;\;F=(\lambda_{2})I_{d_{1}}$

$C_{00}={\left[\begin{array}{cc} I_{a-1}⨂Z_{1} & C_{0}^{\prime }\\ 0 & Z_{2} \end{array}\right]}_{d_{1}\times d_{1}};\;\;C_{0}^{\prime }={\left[\begin{array}{c} \gamma_{1}I_{b}\\ \vdots \\ \gamma_{1}I_{b} \end{array}\right]}_{b(S_{1}+1-s_{1})\times b};\;A_{3}={\left[\begin{array}{cc} H_{1} & 0\\ H_{1} & 0 \end{array}\right]}_{d_{3}\times d_{3}}$

$Z_{1}={\left[\begin{array}{cc} (-\lambda_{2}-\lambda_{1}-\gamma_{2}-\gamma_{1})I_{b-1} & \gamma_{1}e\\ 0 & \left(-\lambda_{2}-\lambda_{1}-\gamma_{1}\right) \end{array}\right]}_{b\times b}$

$Z_{3}={\left[\begin{array}{cc} (-\lambda_{2}-\lambda_{1}p-\gamma_{2}-\gamma_{1}-\mu_{2})I_{b-1} & \gamma_{1}e\\ 0 & \left(-\lambda_{2}-\lambda_{1}p-\gamma_{1}-\mu_{2}\right) \end{array}\right]}_{b\times b}$

$H_{1}={\left[\begin{array}{ccccc} H & 0 & 0 & ... & 0 \end{array}\right]}_{d_{1}\times d_{2}}^{T}$

$D={\left[\begin{array}{cc} I_{a-1}⨂Z_{3} & C_{0}^{\prime }\\ 0 & Z_{4} \end{array}\right]}_{d_{1}\times d_{1}}\;;\;\;\;G=\mu_{2}I_{d_{1}};\;\;Z_{2}=Z_{1}+\gamma_{1}I_{b}\;;\;\;\;\;Z_{4}=Z_{3}+\gamma_{1}I_{b}$$D_{1}=D+\lambda_{2}I_{d_{1}};\;\;D_{2}=D-C_{1}+C_{2}\;\;;A_{21}$ is a zero matrix of order $d_{2}\times d_{2}$

$A_{11}={\left[\begin{array}{cccccc} D_{2} & F & 0 & ... & 0 & 0\\ 0 & D & F & ... & 0 & 0\\ \vdots \\ 0 & 0 & 0 & ... & D & F\\ 0 & 0 & 0 & ... & 0 & D_{1} \end{array}\right]}_{d_{2}\times d_{2}};\;\;\;A_{12}={\left[\begin{array}{cc} 0 & 0\\ \mathrm{GI} & 0 \end{array}\right]}_{d_{2}\times d_{2}}$

$A_{22}^{\prime }={\left[\begin{array}{ccccccc} D & F & 0 & ... & 0 & 0 & 0\\ G & D & F & ... & 0 & 0 & 0\\ \vdots \\ 0 & 0 & 0 & ... & G & D & F\\ 0 & 0 & 0 & ... & 0 & G & D_{1} \end{array}\right]}_{d_{2}\times d_{2}};\;\;\;A_{22}={\left[\begin{array}{ccccccc} D_{2} & F & 0 & ... & 0 & 0 & 0\\ G & D & F & ... & 0 & 0 & 0\\ \vdots \\ 0 & 0 & 0 & ... & G & D & F\\ 0 & 0 & 0 & ... & 0 & G & D_{1} \end{array}\right]}_{d_{2}\times d_{2}}$;

$A_{2}^{\prime }={\left[\begin{array}{cc} A_{11} & A_{12}\\ A_{21} & A_{22} \end{array}\right]}_{d_{3}\times d_{3}};\;\;A_{2}={\left[\begin{array}{cc} A_{11} & A_{12}\\ A_{21} & A_{22} \end{array}\right]}_{d_{3}\times d_{3}}$

### 3.2 Steady state analysis

To get the stability condition, we examine the Markov chain $\left\{X(t),t\geq 0\}=\{(N_{2}(t),I_{1}(t),I_{2}(t),S(t));t\geq 0\right\}$ on the finite state space $\left\{(0,i_{1},i_{2},0);\;i_{1}=s_{1},...,S_{1},S_{1}^{⁎};i_{2}=s_{2},...,S_{2},S_{2}^{⁎}\}⋃\{(n_{2},i_{1},i_{2},1);\;i_{1}=s_{1},...,S_{1},S_{1}^{⁎};\;i_{2}=s_{2},...,S_{2},S_{2}^{⁎};\;0\leq n_{2}\leq M;\;M\geq 1\}⋃\{(n_{2},i_{1},i_{2},2);\;i_{1}=s_{1},...,S_{1},S_{1}^{⁎};\;i_{2}=s_{2},...,S_{2},S_{2}^{⁎};\;0\leq n_{2}\leq M\;;\;M\geq 1\right\}$.

Let $\Phi =(\Phi_{0},\Phi_{1},\Phi_{2},...,\;\Phi_{b-1})$, where $b=\frac{d_{3}}{d_{1}}=2(M+1)$ denote the steady state probability vector of this Markov chain, and $\Phi_{i}=(\Phi_{i}(1),\Phi_{i}(2),\Phi_{i}(3),...,\;\Phi_{i}(d_{1}))$. Infinitesimal generator of {X(t),t≥0} is $A=A_{1}+A_{2}+A_{3}$ where

$A={\left[\begin{array}{cccccccc} D_{2}+E+H & F & 0 & 0...\;\;\;0 & 0 & 0 & 0...\;\;\;0 & 0\\ 0 & D+E & F & 0...\;\;\;0 & G & 0 & 0...\;\;\;0 & 0\\ 0 & 0 & 0 & 0...\;\;\;F & 0 & 0 & G...\;\;\;0 & 0\\ 0 & 0 & 0 & 0...\;\;\;D_{1}+E & 0 & 0 & 0...\;\;\;G & 0\\ H & 0 & 0 & 0...\;\;\;0 & D_{2}+E & F & 0...\;\;\;0 & 0\\ 0 & 0 & 0 & 0...\;\;\;0 & G & D+E & F...\;\;\;0 & 0\\ 0 & 0 & 0 & 0...\;\;\;0 & 0 & 0 & 0...\;\;\;D+E & F\\ 0 & 0 & 0 & 0...\;\;\;0 & 0 & 0 & 0...\;\;\;G & D_{1}+E \end{array}\right]}_{d_{3}\times d_{3}}$

Then Φ satisfies

(1) ΦA=0 and Φe=1

where **e** is a column vector of 1's. Substituting the values of Φ and **A** in equation (1), we get the following sets of equations.

(2) $$\left\{ \begin{array}{cc} \Phi_{0}(D_{2}+E+H)+\Phi_{\frac{b}{2}}H & =0\\ \Phi_{0}F+\Phi_{1}\left(D+E\right) & =0\\ \Phi_{i}F+\Phi_{i+1}\left(D+E\right) & =0,\;\text{for }i=1,2,\ldots ,\left(\frac{b}{2}-3\right)\\ \Phi_{\frac{b}{2}-2}F+\Phi_{\frac{b}{2}-1}\left(D+E\right){(D}_{1}+E) & =0 \end{array}\right.$$

(3) $$\left\{ \begin{array}{c} \Phi_{1}G\;\;+\Phi_{\frac{b}{2}}(D_{2}+E)\;\;+\Phi_{\frac{b}{2}+1}G\;\;=0\\ \Phi_{2}G\;\;+\Phi_{\frac{b}{2}}F\;\;\;+\Phi_{\frac{b}{2}+1}\left(D+E\right)\;\;+\Phi_{\frac{b}{2}+2}G\;\;=0\\ \Phi_{i}G\;\;+\Phi_{\frac{b}{2}+(i-2)}F\;\;+\Phi_{\frac{b}{2}+(i-1)}\left(D+E\right)\;\;+\Phi_{\frac{b}{2}+i}G\;\;=0,\;\;i=3,4,...,(\frac{b}{2}-1)\\ \Phi_{\left(b-2\right)}F\;\;+\;\;\Phi_{\left(b-1\right)}(D_{1}+E)\;\;=0 \end{array}\right.$$

From the set of equations (2), we get

$\Phi_{i}\;=\;{\left(-1\right)}^{i}\;\Phi_{0}{\left[F{\left(D+E\right)}^{-1}\right]}^{i}\;,\;\;\;i=1,2,...,(\frac{b}{2}-2)$

$\Phi_{\frac{b}{2}-1}={\left(-1\right)}^{\left(\frac{b}{2}-1\right)}\;\Phi_{0}{\left[F{\left(D+E\right)}^{-1}\right]}^{\left(\frac{b}{2}-2\right)}[F{\left(D_{1}+E\right)}^{-1}]$

From the set of equations (3), we get

$\Phi_{\frac{b}{2}}=\sum_{j=1}^{\frac{b}{2}-1}\;{\left(-1\right)}^{\frac{b}{2}-j}\;\Phi_{\frac{b}{2}-j}\;\Pi_{r=j+1}^{\frac{b}{2}}G\overset{ˆ}{\overset{ˆ}{G_{r}}}$

where $\overset{ˆ}{\overset{ˆ}{G_{1}}}=F{\left(D_{1}+E\right)}^{-1}\;\;;\;\overset{ˆ}{\overset{ˆ}{G_{2}}}={\left[\left(D+E\right)-\overset{ˆ}{\overset{ˆ}{G_{1}}}G\right]}^{-1}$

$\overset{ˆ}{\overset{ˆ}{G_{i}}}={\left[(D+E)-F\;{\overset{ˆ}{\overset{ˆ}{G}}}_{\left(i-1\right)}G\right]}^{-1},\;\;i=3,4,...,(\frac{b}{2}-1)\;\;;\;\;\text{and}$

$\overset{ˆ}{\overset{ˆ}{G_{\frac{b}{2}}}}={\left[(D_{2}+E)-F{\overset{ˆ}{\overset{ˆ}{G}}}_{\left(i-1\right)}G\right]}^{-1}$

Substituting values of $\Phi_{1},\Phi_{2},...,\Phi_{\frac{b}{2}-1}$ and $\Phi_{\frac{b}{2}}$ in equations of set (3) we get the values of $\Phi_{\frac{b}{2}+1},...,\Phi_{b-2},\Phi_{b-1}$ in terms of $\Phi_{0}$. Thus

$\Phi_{\frac{b}{2}+1}=\;-[\Phi_{1}G\;+\Phi_{\frac{b}{2}}(D_{2}+E)]G^{-1}$

$\Phi_{b/2+i}=\;-[\Phi_{i}G\;+\Phi_{\frac{b}{2}+(i-2)}F\;+\Phi_{\frac{b}{2}+(i-1)}\left(D+E\right)]G^{-1},\;\;i=2,3,...,(\frac{b}{2}-1)$

Thus we get all values of $\Phi_{i},i=1,2,...,(b-1)$ in terms of $\Phi_{0}$. Then from the normalizing condition Φe=1, we get the value of $\Phi_{0}$. The following lemma establishes the stability condition of the queueing inventory system under study.

### 3.3 Stability condition

Lemma 3.3.1 *The queueing inventory system described in Section-3 is stable if and only if*

(4) $$\lambda_{1}p<(\Phi_{0}+\Phi_{b/2})\mu_{1}e,$$

*The terms* $\Phi_{0}$ *and* $\Phi_{b/2}$ *were defined in the previous section and* **e** *is a column vector of 1's of corresponding order.*

Proof The LIQBD description of the model shows that the queueing inventory system is stable ([31]) if and only if $\Phi A_{1}e<\Phi A_{3}e$. From the value of Φ computed from the previous section we can see that this condition reduces to (4), which indicates that the rate at which customers joining the pool should be less than the service rate of the non priority customers. □

### 3.4 Steady-state probability vector

Assuming that the stability condition in section 3.3 is satisfied, we give an outline of the computation of the steady state probability vector of the system. Let **x** denote the steady state probability vector of the generator **Q**. Then **x** satisfies the condition

(5) x.Q=0withx.e=1

Partition **x** as $\text{x}=(\;{\text{x}}_{0},\;{\text{x}}_{1},\;{\text{x}}_{2},...)$. Then the subvectors in **x** are, ${\text{x}}_{n_{1}}=\{x_{n_{1}}(0,i_{1},i_{2},0);i_{1}=s_{1},...,S_{1},S_{1}^{⁎};\;i_{2}=s_{2},...,S_{2},S_{2}^{⁎}\}⋃\{x_{n_{1}}(n_{2},i_{1},i_{2},1);\;i_{1}=s_{1},...,S_{1},S_{1}^{⁎};i_{2}=s_{2},...,S_{2},S_{2}^{⁎};0\leq n_{2}\leq M;\;M\geq 1\}⋃\{x_{n_{1}}(n_{2},i_{1},i_{2},2);i_{1}=s_{1},...,S_{1},S_{1}^{⁎};\;i_{2}=s_{2},...,S_{2},S_{2}^{⁎};\;0\leq n_{2}\leq M;\;M\geq 1\}$, for $n_{1}\geq 0$. We see that, the steady state probability vector $\text{x}=({\text{x}}_{0},{\text{x}}_{1},{\text{x}}_{2},...)$ is given by ${\text{x}}_{n_{1}}={\text{x}}_{1}R^{n_{1}-1}\;\;\;,\;n_{1}\geq 2$

Here **R** is the minimal non-negative solution (see [31]) to the matrix quadratic equation:

$A_{1}+RA_{2}+R^{2}A_{3}=0$ and the vectors ${\text{x}}_{0}$ and ${\text{x}}_{1}$ are given by the boundary equations

${\text{x}}_{0}B_{00}+{\text{x}}_{1}B_{10}=0$

${\text{x}}_{0}B_{01}+{\text{x}}_{1}(A_{2}+RA_{3})=0$

The normalizing condition (5) gives ${\text{x}}_{0}[I-B_{01}{\left(A_{2}+RA_{3}\right)}^{-1}{\left(I-R\right)}^{-1}e]=1$

## 4 System performance measures

In this section, we derive performance measures of the system under the steady state.

1) Expected number of type-I (non priority) customers in the system

$EN_{1}=\sum_{n_{1}=0}^{\infty }\;\;\sum_{n_{2}=0}^{M}\;\;\sum_{i_{2}=s_{2}}^{S_{2}^{⁎}}\;\;\sum_{i_{1}=s_{1}}^{S_{1}^{⁎}}\;\;\sum_{r=0}^{2}\;\;n_{1}x_{n_{1}}(n_{2},i_{1},i_{2},r)$

2) Expected number of type-II (priority) customers in the system

$EN_{2}=\sum_{n_{1}=0}^{\infty }\;\;\sum_{n_{2}=0}^{M}\;\;\sum_{i_{2}=s_{2}}^{S_{2}^{⁎}}\;\;\sum_{i_{1}=s_{1}}^{S_{1}^{⁎}}\;\;\sum_{r=0}^{2}\;\;n_{2}x_{n_{1}}(n_{2},i_{1},i_{2},r)$

3) Expected inventory level of commodity-1

$EI_{1}=\sum_{n_{1}=0}^{\infty }\;\;\sum_{n_{2}=0}^{M}\;\;\sum_{i_{2}=s_{2}}^{S_{2}^{⁎}}\;\;\sum_{i_{1}=s_{1}}^{S_{1}^{⁎}}\;\;\sum_{r=0}^{2}\;\;i_{1}x_{n_{1}}(n_{2},i_{1},i_{2},r)$

4) Expected inventory level of commodity-2

$EI_{2}=\sum_{n_{1}=0}^{\infty }\;\;\sum_{n_{2}=0}^{M}\;\;\sum_{i_{2}=s_{2}}^{S_{2}^{⁎}}\;\;\sum_{i_{1}=s_{1}}^{S_{1}^{⁎}}\;\;\sum_{r=0}^{2}\;\;i_{2}x_{n_{1}}(n_{2},i_{1},i_{2},r)$

5) Expected inventory level of commodity-1 immediately on realization of CLT

$E_{I_{1}}(CLT)=\frac{\gamma_{1}}{\gamma_{1}+\lambda_{1}}\;\sum_{j_{1}=s_{1}}^{S_{1}}\;\;\sum_{j_{2}=s_{2}}^{S_{2}^{⁎}}\;\;j_{1}x_{0}(0,j_{1},j_{2},0)+$

$\frac{\gamma_{1}}{\gamma_{1}+\lambda_{1}+\mu_{1}}\;\sum_{n_{1}=0}^{\infty }\;\;\;\sum_{j_{1}=s_{1}}^{S_{1}}\;\;\sum_{j_{2}=s_{2}}^{S{⁎}_{2}}\;\;j_{1}x_{n_{1}}(0,j_{1},j_{2},1)$

6) Expected inventory level of commodity-2 immediately on realization of CLT

$E_{I_{2}}(CLT)=\frac{\gamma_{2}}{\gamma_{2}+\lambda_{2}}\;\sum_{j_{1}=s_{1}}^{S_{1}^{⁎}}\sum_{j_{2}=s_{2}}^{S_{2}}\;\;j_{2}x_{0}(0,j_{1},j_{2},0)+$

$\frac{\gamma_{2}}{\gamma_{2}+\lambda_{2}+\mu_{2}}\;\sum_{n_{1}=0}^{\infty }\;\;\sum_{n_{2}=0}^{M}\;\;\sum_{j_{1}=s_{1}}^{S_{1}^{⁎}}\;\;\sum_{j_{2}=s_{2}}^{S_{2}}\;\;j_{2}x_{n_{1}}(n_{2},j_{1},j_{2},2)$

7) Probability that the server is busy processing a demand of commodity-1 alone

$P_{C1}=\sum_{n_{1}=1}^{\infty }\;\;\sum_{j_{1}=s_{1}}^{S_{1}^{⁎}}\;\;\sum_{j_{2}=s_{2}}^{S_{2}^{⁎}}\;\;x_{n_{1}}(0,j_{1},j_{2},1)$

8) Probability that the server is busy processing a demand of commodity-2 alone

$P_{C2}=\sum_{n_{1}=0}^{\infty }\;\;\sum_{n_{2}=1}^{M}\;\;\sum_{j_{1}=s_{1}}^{S_{1}^{⁎}}\;\;\sum_{j_{2}=s_{2}}^{S_{2}^{⁎}}\;\;x_{n_{1}}(n_{2},j_{1},j_{2},2)$

9(a) Probability that the server is idle

$P_{\left(serveridle\right)}=\sum_{j_{1}=s_{1}}^{S_{1}^{⁎}}\;\;\sum_{j_{2}=s_{2}}^{S_{2}^{⁎}}\;\;x_{0}(0,j_{1},j_{2},0)$

(b) Probability that the server is busy

$P_{\left(Serverbusy\right)}=\sum_{n_{1}=0}^{\infty }\;\;\sum_{n_{2}=0}^{M}\;\;\sum_{j_{1}=s_{1}}^{S_{1}^{⁎}}\;\;\sum_{j_{2}=s_{2}}^{S_{2}^{⁎}}\;\;x_{n_{1}}(n_{2},j_{1},j_{2},1)+$

$\;\;\;\;\sum_{n_{1}=0}^{\infty }\;\;\sum_{n_{2}=1}^{M}\;\;\sum_{j_{1}=s_{1}}^{S_{1}^{⁎}}\;\;\sum_{j_{2}=s_{2}}^{S_{2}^{⁎}}\;\;x_{n_{1}}(n_{2},j_{1},j_{2},2)$

10) Probability that commodity-1 alone reaches its reorder level before realizing CLT

$P_{C1_{S_{1}}}=\sum_{n_{1}=1}^{\infty }\;\;\sum_{n_{2}=0}^{M}\;\;\sum_{j_{2}=s_{2}}^{S_{2}^{⁎}}\;\;x_{n_{1}}(n_{2},s_{1},j_{2},1)$

11) Probability that commodity-2 alone reaches its reorder level before realizing CLT

$P_{C2_{S_{2}}}=\sum_{n_{1}=0}^{\infty }\;\;\sum_{n_{2}=1}^{M}\;\;\sum_{j_{1}=s_{1}}^{S_{1}^{⁎}}\;\;x_{n_{1}}(n_{2},j_{1},s_{2},2)$

12(a) Expected reorder rate of commodity-1 before realizing CLT

$\psi_{1}=\mu_{1}\;\sum_{n_{1}=0}^{\infty }\;\;\sum_{n_{2}=0}^{M}\;\;\sum_{j_{2}=s_{2}}^{S_{2}^{⁎}}\;\;x_{n_{1}}(n_{2},s_{1},j_{2},1)$

12(b) Total Expected reorder rate of commodity-1

$E_{R_{1}}$ = $\psi_{1}+\frac{\gamma_{1}^{2}}{\lambda_{1}+\gamma_{1}+\mu_{1}}\;\sum_{n_{1}=0}^{\infty }\;\sum_{j_{1}=s_{1}}^{S_{1}}\;\sum_{j_{2}=s_{2}}^{S_{2}^{⁎}}\;\sum_{n_{2}=0}^{M}\;\;\sum_{r=0}^{2}\;\;x_{n_{1}}(n_{2},j_{1},j_{2},r)$

12(c) Average number of reorders due to realization of CLT of commodity-1 in a cycle

$\sigma_{1}$ = $(E_{R_{1}}-\psi_{1})\eta_{1}$, where $\eta_{1}$ is the expected cycle length given in sec-3.3.

13(a) Expected reorder rate of commodity-2 before realizing CLT

$\psi_{2}=\mu_{2}\;\sum_{n_{1}=0}^{\infty }\;\;\sum_{n_{2}=0}^{M}\;\;\sum_{j_{1}=s_{1}}^{S_{1}^{⁎}}\;\;x_{n_{1}}(n_{2},j_{1},s_{2},2)$

13(b) Expected reorder rate of commodity-2

$E_{R_{2}}$ = $\psi_{2}+\frac{\gamma_{2}^{2}}{\lambda_{2}+\gamma_{2}+\mu_{2}}\;\sum_{n_{1}=0}^{\infty }\;\sum_{j_{1}=s_{1}}^{S_{1}^{⁎}}\;\sum_{j_{2}=s_{2}}^{S_{2}}\;\;\sum_{n_{2}=0}^{M}\;\;\sum_{r=0}^{2}\;\;x_{n_{1}}(n_{2},j_{1},j_{2},r)$

13(c) Average number of reorders due to realization of CLT of commodity-2 in a cycle

$\sigma_{2}$ = $(E_{R_{2}}-\psi_{2})⁎\eta_{2}$, where $\eta_{2}$ is the expected cycle length given in sec-3.3.

14(a) Expected loss rate of priority customers in the system

$EL_{2}=\lambda_{2}\sum_{n_{1}=0}^{\infty }\;\;\sum_{j{}_{1}=s{}_{1}}^{S_{1}^{⁎}}\;\;\sum_{j_{2}=s_{2}}^{S_{2}^{⁎}}\;\;x_{n_{1}}(M,j_{1},j_{2},2)$

14(b) Expected loss rate of non priority customers in the system

$EL_{1}=\lambda_{1}(1-p)\sum_{n_{1}=0}^{\infty }\;\;\sum_{j{}_{1}=s{}_{1}}^{S_{1}^{⁎}}\;\;\sum_{j_{2}=s_{2}}^{S_{2}^{⁎}}\;\sum_{n_{2}=0}^{M}\;\sum_{r=1}^{2}\;\;x_{n_{1}}(n_{2},j_{1},j_{2},r)$

### 4.1 Average sojourn time of a non-priority (type-1) customer

In this section, we calculate the average sojourn time of a type-1 customer in the pool. We consider the Markov process $W_{1}(t)=\{(N_{3}(t),N_{2}(t),I_{1}(t),I_{2}(t),S(t));\;t\geq 0\}$, where $N_{3}(t)$ is the rank of the tagged non-priority customer in the pool at time *t*. The rank of a customer is defined as *r* if that customer is the $r^{th}$ customer in the pool at time *t* and his rank decreases as and when the customers ahead of him leave the system. The customers join after the marked customer cannot change the rank, the transitions in $W_{1}(t)$ are only to one side of the diagonal of the rate matrix. The state space of $\left\{W_{1}(t),t\geq 0\right\}$ is $\left\{(n_{3},n_{2},i_{1},i_{2},1);\;1\leq n_{3}\leq r\;;s_{1}\leq i_{1}\leq S_{1},S_{1}^{⁎};\;0\leq n_{2}\leq M;\;s_{2}\leq i_{2}\leq S_{2},S_{2}^{⁎};\;\;M\geq 1\}⋃\{(n_{3},n_{2},i_{1},i_{2},2);\;1\leq n_{3}\leq r;\;s_{1}\leq i_{1}\leq S_{1},S_{1}^{⁎};\;0\leq n_{2}\leq M;\;s_{2}\leq i_{2}\leq S_{2},S_{2}^{⁎};\;M\geq 1\}⋃\{{△}_{1}\right\}$, where $\left\{{△}_{1}\right\}$ is the absorbing state indicating that the type-1 customer is selected for service. Thus the infinitesimal generator of $\left\{W_{1}(t);t\geq 0\right\}$ is given by $W_{1}=\left[\begin{array}{cc} T_{1}^{△} & T_{1}^{{△}^{0}}\\ 0 & 0 \end{array}\right]$ where

$T_{1}^{△}={\left[\begin{array}{ccccc} N & N_{1}\\ & N & N_{1}\\ & & \ddots & \ddots \\ & & & N & N_{1}\\ & & & & N \end{array}\right]}_{rd_{3}\times rd_{3}};\;\;T_{1}^{{△}^{0}}={\left[\begin{array}{c} 0\\ 0\\ 0\\ \vdots \\ N_{3} \end{array}\right]}_{rd_{3}\times 1}$

$N_{1}=A_{3}$; $N_{2}={\left[\begin{array}{c} N_{2}^{\prime }\\ 0 \end{array}\right]}_{d_{2}\times 1};\;N_{3}={\left[\begin{array}{c} N_{2}\\ N_{2} \end{array}\right]}_{d_{3}\times 1};\;\;D_{2}^{△}=D_{2}+E\;;\;\;D_{1}^{△}=D_{1}+E$;

$D^{△}=D+E\;\;;\;\;\;N_{2}^{\prime }$ is of order $d_{1}\times 1$, all its elements are $\mu_{1}$ and

$N={\left[\begin{array}{cccccccccc} D_{2}^{△} & F & 0 & 0... & 0 & 0 & 0 & 0... & 0 & 0\\ 0 & D^{△} & F & 0... & 0 & G & 0 & 0... & 0 & 0\\ \vdots \\ 0 & 0 & 0 & 0... & F & 0 & 0 & G... & 0 & 0\\ 0 & 0 & 0 & 0... & D_{1}^{△} & 0 & 0 & 0... & G & 0\\ 0 & 0 & 0 & 0... & 0 & D_{2}^{△} & F & 0... & 0 & 0\\ 0 & 0 & 0 & 0... & 0 & G & D^{△} & F... & 0 & 0\\ \vdots \\ 0 & 0 & 0 & 0... & 0 & 0 & 0 & 0... & D^{△} & F\\ 0 & 0 & 0 & 0... & 0 & 0 & 0 & 0... & G & D_{1}^{△} \end{array}\right]}_{d_{3}\times d_{3}}$

Now, the sojourn time of a marked customer, who joins the queue as the $r^{th}$ customer is the time until absorption of the Markov Chain $\left\{W_{1}(t),t\geq 0\right\}$. The random variable $W_{1}(t)$ follows phase type distribution. Thus the average waiting time of the $r^{th}$ customer is a column vector $E_{T_{1r}}=-\{T_{1}^{{△}^{-}1}e,r\geq 1\}$

Therefore the average waiting time of a general (type-I) customer in the system is

$Ew_{1}=\sum_{n_{1}=0}^{\infty }\sum_{r=1}^{\infty }\;x_{n_{1}}(r)E_{T_{1r}}$, where $x_{n_{1}}(r)$ is a row vector of dimension $rd_{3}$ and $x_{n_{1}}(r)=\{(r,m,i_{1},i_{2},l),(r-1,m,i_{1},i_{2},l),...,(1,m,i_{1},i_{2},l);\;0\leq m\leq M;\;s_{1}\leq i_{1}\leq S_{1},S_{1}^{⁎};\;s_{2}\leq i_{2}\leq S_{2},S_{2}^{⁎};l=1,2\}$

### 4.2 Expected sojourn time of a priority (type-2) customer

Here, we calculate the average sojourn time of a priority customer. In this case when at least one customer in the buffer, the server continuously serve customers from the buffer until the buffer becomes empty. To calculate the average sojourn time of a type-2 customer, we examine the Markov process $W_{2}(t)=\{(N(t),I_{2}(t));t\geq 0\}$, where N(t) is the rank of the tagged customer in the buffer at time *t*. The rank N(t) of the customer is *r* (finite) if he is the $r^{th}$ customer in the queue at time *t*. His rank decreases to 1 as the customers ahead of him leave the system after completing service. The state space of the process is $\left\{(n,i_{2}):1\leq n\leq r;\;r\leq M,s_{2}\leq i_{2}\leq S_{2},S_{2}^{{}^{⁎}}\}⋃\{{△}_{2}\right\}$, where $\left\{{△}_{2}\right\}$ is the absorbing state which shows that the marked customer entered in to service. The rate matrix of $\left\{W_{2}(t);t\geq 0\right\}$ is given by

$W_{2}=\left[\begin{array}{cc} T_{2} & T_{2}^{0}\\ 0 & 0 \end{array}\right]$ where $T_{2}={\left[\begin{array}{ccccc} M_{2} & M_{1}\\ & M_{2} & M_{1}\\ & & \ddots & \ddots \\ & & & M_{2} & M_{1}\\ & & & & M_{2} \end{array}\right]}_{rb\times rb}$; $T_{2}^{0}={\left[\begin{array}{c} 0\\ 0\\ 0\\ \vdots \\ M_{3} \end{array}\right]}_{rb\times 1}$

$M_{1}=\mu_{2}I_{b}\;;\;\;\;M_{3}$ is a column vector of order b×1 whose elements are $\mu_{2}$ and $M_{2}$ is a square matrix of order *b*.

$M_{2}=\left\{ \begin{array}{cc} -\gamma_{2}-\mu_{2}, & i_{2}:s_{2},s_{2}+1...S_{2},i_{2}=j_{2}\\ -\mu_{2}, & i_{2}=S_{2}^{⁎};i_{2}=j_{2}\\ \gamma_{2}, & i_{2}=s_{2}...S_{2};j_{2}=S_{2}^{⁎} \end{array}\right.\;$

Now, the waiting time of type-2 customer, who enters the queue as the $r^{th}$ customer is the time until absorption of the Markov chain $\left\{W_{2}(t);t\geq 0\right\}$. The random variable $W_{2}(t)$ follows phase type distribution. Thus the average sojourn time of the $r^{th}$ customer is a column vector $E_{T_{2r}}=-T_{2}^{-1}e$. Therefore the average waiting time of a general (type-2) customer in the system is $Ew_{2}$ = $\sum_{n_{1}=0}^{\infty }\sum_{r=1}^{M}y_{n_{1}}(r)E_{T_{2}r}$, where $y_{n_{1}}(r)$ is a row vector of dimension $r(S_{2}-s_{2}+2)$ and is given by $y_{n_{1}}(r)=\{(r,s_{2}),(r,s_{2}+1),...,(r,S_{2}),(r,S_{2}^{⁎}),(r-1,s_{2}),(r-1,s_{2}+1),...,(r-1,S_{2^{⁎}}),...,(1,s_{2}),(1,s_{2}+1),...,(1,S_{2}),(1,S_{2}^{⁎})\}$

### 4.3 Analysis of cycle time for commodity-1

A cycle time for commodity-1 is defined as the time until replenishment of inventory starting from the maximum inventory level. That is, duration between two consecutive $S_{1}$ to $S_{1}$ or $S_{1}^{⁎}$ transitions. That is, completion of a cycle and the starting of the next cycle can be either due to realization of common lifetime or by a service completion when there are $s_{1}$ items left in the inventory, which ever occurs first. For the computation of the expected duration of a cycle, we assume that the pool capacity is K>0 (sufficiently large); Now examine the Markov chain $\left\{(N_{4}(t),N_{2}(t),I_{1}(t),I_{2}(t),S(t));t\geq 0\right\}$ over the finite state space $\left\{(n_{4},n_{2},j_{1},j_{2},r):0\leq n_{4}\leq K\;;0\leq n_{2}\leq M\;;M\geq 1\;;s_{1}\leq j_{1}\leq S_{1},S_{1}^{⁎}\;;s_{2}\leq j_{2}\leq S_{2},S_{2}^{⁎}\;;1\leq r\leq 2\}⋃\{(0,0,j_{1},j_{2},0):s_{1}\leq j_{1}\leq S_{1},S_{1}^{⁎}\;;s_{2}\leq j_{2}\leq S_{2},S_{2}^{⁎}\}⋃\{{△}_{\mu_{1}}\}⋃\{{△}_{CLT_{1}}\right\}$.

The symbol $N_{4}(t)$ is the number of customers in the finite pool, $\left\{{△}_{\mu_{1}}\right\}$ is an absorbing state consequent to the replenishment order placed for commodity-1 and ${△}_{CLT_{1}}$ is an absorbing state consequent to the replenishment order due to realization of common lifetime of commodity-1 and all other terms are defined in section 3.1. Let $d_{2}^{\prime }=(K+1)d_{2}$.

The infinitesimal generator of $\left\{W_{3}(t);t\geq 0\right\}$ is given by

$W_{3}=\left[\begin{array}{ccc} \overset{ˆ}{T} & {\overset{ˆ}{T}}_{\mu_{1}}^{0} & {\overset{ˆ}{T}}_{CLT_{1}}^{0}\\ 0 & 0 & 0 \end{array}\right]$. The matrices in the generator are defined as follows:

$\overset{ˆ}{T}=\left[\begin{array}{cccccc} G_{00}^{\prime } & G_{01}^{\prime }\\ & G_{1}^{\prime } & G_{0}^{\prime }\\ & & \ddots & \ddots & & \\ & & & G_{1}^{\prime } & G_{0}^{\prime }\\ & & & & G_{1}^{\prime \prime } \end{array}\right]\;\;\;;\;\;{\overset{ˆ}{T}}_{\mu_{1}}^{0}={\left[\begin{array}{c} G_{3}^{\prime }\\ G_{5}^{\prime }\\ G_{5}^{\prime }\\ \vdots \\ G_{5}^{\prime } \end{array}\right]}_{(2d_{2}^{\prime }+d_{1})\times 1};\;\;\;G_{3}^{\prime }={\left[\begin{array}{c} 0\\ G_{31}^{\prime }\\ 0\\ \vdots \\ 0 \end{array}\right]}_{(d_{3}+d_{1})\times 1}$

$G_{01}^{\prime }=\left[\begin{array}{c} 0\\ A_{1} \end{array}\right]$; $G_{5}^{\prime }={\left[\begin{array}{cccccccc} G_{31}^{\prime } & 0 & ... & 0 & G_{31}^{\prime } & 0 & ... & 0 \end{array}\right]}_{1\times d_{3}}^{T}$

$G_{01}^{\prime }$ is a matrix of order $(d_{3}+d_{1})\times d_{3}$. $G_{00}^{\prime }$ is a square matrix of order $(d_{3}+d_{1}),G_{0}^{\prime },G_{1}^{\prime },G_{1}^{\prime \prime }$ are square matrices of order $d_{3},\overset{ˆ}{T}$ is a square matrix of order $2d_{2}^{\prime }+d_{1}$. $T_{CLT}^{0}$ is a column vector of $\gamma_{1}$ of order $(2d_{2}^{\prime }+d_{1})\times 1$, $G_{31}^{\prime }$ is a column vector of $\mu_{1}$ of order b×1,

$G_{00}^{\prime }=\left[\begin{array}{cc} C_{00}^{\prime } & C_{01}\\ C_{02}^{\prime } & {\overset{ˆ}{A}}_{2}^{\prime } \end{array}\right]$; $C_{02}^{\prime }={\left[\begin{array}{ccccccccc} 0 & 0 & ... & 0 & C_{2} & 0 & 0 & ... & 0 \end{array}\right]}_{d_{1}\times d_{3}}^{T}$

$C_{00}^{\prime }={\left[\begin{array}{cc} I_{a-1}⨂Z_{1} & 0\\ 0 & Z_{2} \end{array}\right]}_{d_{1}\times d_{1}}$; $G_{1}^{\prime \prime }=G_{1}^{\prime }+G_{0}^{\prime }\;;G_{0}^{\prime }=A_{1}\;;G_{1}^{\prime }={\overset{ˆ}{A}}_{2}$

${\overset{ˆ}{A}}_{11}={\left[\begin{array}{cccccc} {\overset{ˆ}{D}}_{2} & F & 0 & ... & 0 & 0\\ 0 & \overset{ˆ}{D} & F & ... & 0 & 0\\ \vdots \\ 0 & 0 & 0 & ... & \overset{ˆ}{D} & F\\ 0 & 0 & 0 & ... & 0 & {\overset{ˆ}{D}}_{1} \end{array}\right]}_{d_{2}\times d_{2}};\;\;\;\;{\overset{ˆ}{A}}_{22}^{\prime }={\left[\begin{array}{ccccccc} \overset{ˆ}{D} & F & 0 & ... & 0 & 0 & 0\\ G & \overset{ˆ}{D} & F & ... & 0 & 0 & 0\\ \vdots \\ 0 & 0 & 0 & ... & G & \overset{ˆ}{D} & F\\ 0 & 0 & 0 & ... & 0 & G & {\overset{ˆ}{D}}_{1} \end{array}\right]}_{d_{2}\times d_{2}}$

${\overset{ˆ}{A}}_{22}={\left[\begin{array}{ccccccc} {\overset{ˆ}{D}}_{2} & F & 0 & ... & 0 & 0 & 0\\ G & \overset{ˆ}{D} & F & ... & 0 & 0 & 0\\ \vdots \\ 0 & 0 & 0 & ... & G & \overset{ˆ}{D} & F\\ 0 & 0 & 0 & ... & 0 & G & {\overset{ˆ}{D}}_{1} \end{array}\right]}_{d_{2}\times d_{2}}\;;\;\;{\overset{ˆ}{A}}_{2}^{\prime }={\left[\begin{array}{cc} {\overset{ˆ}{A}}_{11} & A_{12}\\ A_{21} & {\overset{ˆ}{A}}_{22}^{\prime } \end{array}\right]}_{d_{3}\times d_{3}};\;\;{\overset{ˆ}{A}}_{2}={\left[\begin{array}{cc} {\overset{ˆ}{A}}_{11} & A_{12}\\ A_{21} & {\overset{ˆ}{A}}_{22} \end{array}\right]}_{d_{3}\times d_{3}}$

$\overset{ˆ}{D}={\left[\begin{array}{cc} I_{a-1}⨂Z_{3} & 0\\ 0 & Z_{4} \end{array}\right]}_{d_{1}\times d_{1}}\;\;{\overset{ˆ}{D}}_{2}=\overset{ˆ}{D}-C_{1}+C_{2}\;;\;{\overset{ˆ}{D}}_{1}=\overset{ˆ}{D}+\lambda_{2}I_{d_{1}}$

(a) The distribution of the cycle time of commodity-1 is of phase type with representation $\left(\alpha_{1},\overset{ˆ}{T}\right)$, where $\alpha_{1}=(\alpha_{S_{1}},0,0,...0),\alpha_{S_{1}}=\{(Cx_{0}(0,S_{1},j_{2},0),Cx_{1}(n_{2},S_{1},j_{2},r),...,Cx_{K}(n_{2},S_{1},j_{2},r)$; $s_{2}\leq j_{2}\leq S_{2},S_{2}^{⁎},0\leq n_{2}\leq M;r=1,2\}$ and $C={\left[\sum_{n_{1}=0}^{K}\;\;\sum_{n_{2}=0}^{M}\;\;\sum_{i_{2}=s_{2}}^{S_{2}^{⁎}}\;\;\sum_{r=1}^{2}x_{n_{1}}(n_{2},S_{1},j_{2},r)+\sum_{j_{2}=s_{2}}^{S_{2}^{⁎}}\;\;x_{0}(0,S_{1},j_{2},0)\right]}^{-1}$

(b)The mean cycle length of commodity-1 is $\eta_{1}=-\alpha_{1}{\overset{ˆ}{T}}^{-1}e$

(c) Probability that the inventory level reaches the reorder level $s_{1}$ before realization of CLT of commodity-1 = $-\alpha_{S_{1}}{\overset{ˆ}{T}}^{-1}\;{\overset{ˆ}{T}}_{\mu }^{0}$

(d)Probability that commodity-1 realizes common lifetime before inventory level reaches $s_{1}\;=\;-\alpha_{S_{1}}{\overset{ˆ}{T}}^{-1}\;{\overset{ˆ}{T}}_{CLT}^{0}$

### 4.4 Analysis of cycle time for commodity-2

We compute the cycle time for commodity-2 in a similar way as that of the cycle time for commodity-1. A cycle time of commodity-2 is defined as the time until replenishment of inventory starting from the maximum inventory level. That is, duration between two consecutive $S_{2}$ to $S_{2}$ or $S_{2}^{⁎}$ transitions. That is, the completion of a cycle and starting of the next cycle can be either due to realization of common life time or by a service completion when there are $s_{2}$ items left in the inventory, which ever occurs first. Now examine the Markov chain $\left\{(N_{4}(t),N_{2}(t),I_{1}(t),I_{2}(t),S(t));t\geq 0\right\}$ over the finite state space $\{(n_{4},n_{2},i_{1},i_{2},r);0\leq n_{4}\leq K\;;s_{1}\leq i_{1}\leq S_{1},S_{1}^{⁎}\;;0\leq n_{2}\leq M\;;s_{2}\leq i_{2}\leq S_{2},S_{2}^{⁎}\;;M\geq 1\;;1\leq r\leq 2\}⋃\{(0,0,i_{1},i_{2},0);s_{1}\leq i_{1}\leq S_{1},S_{1}^{⁎}\;;s_{2}\leq i_{2}\leq S_{2},S_{2}^{⁎}\;;M\geq 1\}⋃{△}_{\mu_{2}}⋃{△}_{CLT_{2}}$. All other terms are defined as in section-4.3. Where ${△}_{\mu_{2}}$ is an absorbing state consequent to the replenishment order placed for commodity-2 and ${△}_{CLT_{2}}$ is an absorbing state consequent to the replenishment order due to realization of common lifetime of commodity-2. The infinitesimal generator of this chain is $W_{4}=\left[\begin{array}{ccc} \;\overset{ˆ}{\overset{ˆ}{T}} & {\overset{ˆ}{\overset{ˆ}{T}}}_{\mu_{2}}^{0} & {\overset{ˆ}{\overset{ˆ}{T}}}_{CLT_{2}}^{0}\\ 0 & 0 & 0 \end{array}\right]$; Here the matrices ${\overset{ˆ}{\overset{ˆ}{T}}}_{\mu_{2}}^{0}$ and ${\overset{ˆ}{\overset{ˆ}{T}}}_{CLT_{2}}^{0}$ can be obtained similarly as in the case of commodity-1.

(a) The distribution of the cycle time of commodity-2 is of phase type with representation $\left(\alpha_{2},\overset{ˆ}{\overset{ˆ}{T}}\right)$, where $\alpha_{2}=(\alpha_{S_{2}},0,0,...0),\alpha_{S_{2}}=\{(C^{\prime }x_{0}(0,j_{1},S_{2},0),C^{\prime }x_{1}(n_{2},j_{1},S_{2},r),...,C^{\prime }x_{K}(n_{2},j_{1},S_{2},r))$;

$s_{1}\leq j_{1}\leq S_{1},S_{1}^{⁎};0\leq n_{2}\leq M;\;r=1,2\}$ and $C^{\prime }=[\sum_{i_{1}=s_{1}}^{S_{1}^{⁎}}\;\;x_{0}(0,i_{1},S_{2},0)+$

${\sum_{n_{1}=0}^{K}\;\;\sum_{n_{2}=0}^{M}\;\;\sum_{i_{1}=s_{1}}^{S_{1}^{⁎}}\;\;\sum_{r=1}^{2}\;\;x_{n_{1}}(n_{2},i_{1},S_{2},r)]}^{-1}$

(b) The mean cycle time of commodity-2 is $\eta_{2}=-\alpha_{2}{\overset{ˆ}{\overset{ˆ}{T}}}^{-1}e$

(c) Probability that the inventory level reaches the reorder level $s_{2}$ before realization of CLT of commodity-$2=-\alpha_{S_{2}}{\overset{ˆ}{\overset{ˆ}{T}}}^{-1}\;{\overset{ˆ}{\overset{ˆ}{T}}}_{\mu }^{0}$

(d) Probability that commodity-2 realizes common lifetime before inventory level reaches $s_{2}=-\alpha_{S_{2}}{\overset{ˆ}{\overset{ˆ}{T}}}^{-1}\;{\overset{ˆ}{\overset{ˆ}{T}}}_{CLT}^{0}$

### 4.5 Mean sojourn time in $S_{1}$ before attainment of common lifetime of commodity-1

To evaluate the mean time the system stays with inventory level $S_{1}$, we consider the Markov chain $\left\{(N_{4}(t),N_{2}(t),I_{1}(t),I_{2}(t),S(t));t\geq 0\},N_{4}(t\right)$ is the number of customers in the pool of finite capacity K. Other notations are similar as followed in section-3.1. Its state space is $\{(n_{4},n_{2},i_{1},i_{2},r);0\leq n_{4}\leq K;\;i_{1}=S_{1};\;0\leq n_{2}\leq M;\;s_{2}\leq i_{2}\leq S_{2},S_{2}^{⁎};\;M\geq 1;\;1\leq r\leq 2\}⋃\{(0,0,i_{1},i_{2},0);i_{1}=S_{1};\;s_{2}\leq i_{2}\leq S_{2},S_{2}^{⁎};\;M\geq 1\}⋃{△}_{CLT}$. Here $\left\{{△}_{CLT}\right\}$ is an absorbing state which denotes the attainment of common life time of commodity-1.

The infinitesimal generator of $\left\{W_{S_{1}}(t);t\geq 0\right\}$ is of the form

$W_{S_{1}}=\left[\begin{array}{cc} T_{S1} & T_{S1}^{0}\\ 0 & 0 \end{array}\right]\;\;;\;\;\;\;\;T_{S1}={\left[\begin{array}{ccccc} L_{00} & L_{01}\\ & L_{2} & L_{1}\\ & & \ddots & \ddots \\ & & & L_{2} & L_{1}\\ & & & & L_{2}^{\prime } \end{array}\right]}_{2l_{2}+l_{1}}\;\;;\;\;\;T_{S1}^{0}={\left[\begin{array}{c} \gamma_{1}\\ \gamma_{1}\\ \vdots \\ \gamma_{1} \end{array}\right]}_{(2l_{2}+l_{1})\times 1}$

Where $l_{1}=2(S_{2}-s_{2}+2);\;\;l_{2}=2(S_{2}-s_{2}+2)(K+1)(M+1);\;l_{3}=4(S_{2}-s_{2}+2)(M+1)$ $\overset{ˆ}{d_{1}}=(S_{1}-s_{1}+1)(S_{2}-s_{2}+2)$;

$\overset{ˆ}{d_{2}}=(S_{1}-s_{1}+1)(S_{2}-s_{2}+2)(M+1);\;\;\overset{ˆ}{d_{3}}=2\overset{ˆ}{d_{2}}$

$L_{00}={\left[\begin{array}{cc} C_{00}^{▿} & C_{01}^{▿}\\ C_{02}^{▿} & L_{2}^{▿} \end{array}\right]}_{l_{1}\times l_{3}}\;\;;\;\;\;L_{01}=\left[\begin{array}{c} 0\\ A_{1}^{▿} \end{array}\right]\;\;;\;\;\;{\left[A_{02}^{▿}\right]}_{l_{1}\times l_{1}}=I_{a}⨂J^{▿}\;\;;\;\;\;A_{1}^{▿}=I_{2(M+1)}⨂E^{▿}$

$C_{01}^{▿}={\left[\begin{array}{cccccccccc} 0 & 0 & 0 & ... & 0 & A_{02}^{▿} & 0 & 0 & ... & 0 \end{array}\right]}_{l_{1}\times l_{3}}\;\;;\;\;\;C_{1}^{▿}=\mu_{1}I_{l_{1}}\;\;;\;\;\;E^{▿}=(\lambda_{1}p)I_{l_{1}}$;

$C_{02}^{▿}={\left[\begin{array}{cccccccccc} C_{1}^{▿} & 0 & 0 & ... & 0 & C_{2}^{▿} & 0 & 0 & ... & 0 \end{array}\right]}_{l_{1}\times l_{3}}^{T}\;;\;\;C_{2}^{▿}=\mu_{2}I_{l_{2}}$

The elements of matrix $J^{▿}$ represent the transitions from $\left(0,0,i_{1},i_{2},0\right)$ to $\left(0,0,i_{1},j_{2},2\right)$ where $i_{1},j_{1}$ and $i_{2},j_{2}$ are inventory levels of commodity-1 and commodity-2.

$J^{▿}(0,0,i_{1},i_{2},0)\to (0,0,i_{1},j_{2},2)=\left\{ \begin{array}{c} \lambda_{2},\;\;i_{1}=S_{1};\;i_{2}=s_{2};j_{2}=S_{2}\\ \lambda_{2},\;\;i_{1}=S_{1};\;i_{2}=s_{2}+1,...,S_{2},S_{2}^{⁎};\;\;j_{2}=i_{2}-1\\ 0,\;\;Otherwise \end{array}\right.\;$

$C_{00}^{▿}={\left[\begin{array}{cc} Z_{1}^{▿} & 0\\ 0 & Z_{2}^{▿} \end{array}\right]}_{l_{1}\times l_{1}}\;;\;\;L_{1}=A_{1}^{▿};\;\;D^{▿}={\left[\begin{array}{cc} Z_{3}^{▿} & 0\\ 0 & Z_{4}^{▿} \end{array}\right]}_{l_{3}\times l_{3}}$

$Z_{1}^{▿}(0,0,S_{1},i_{2},0)\to (0,0,S_{1},j_{2},0)=\left\{ \begin{array}{c} -\lambda_{2}-\gamma_{2}-\gamma_{1},\;\;i_{2}=s_{2},...,S_{2};j_{2}=i_{2}\\ -\lambda_{2}-\gamma_{1},\;\;\;\;\;\;i_{2}=S_{2}^{⁎};j_{2}=i_{2}\\ \gamma_{2},\;\;\;\;\;\;\;\;\;\;\;i_{2}=s_{2},...,S_{2};j_{2}=S_{2}^{⁎}\\ 0,\;\;\;\;\;\;\;\;\;Otherwise \end{array}\right.\;$

$Z_{3}^{▿}(0,0,S_{1},i_{2},r)\to (0,0,S_{1},j_{2},r)=\left\{ \begin{array}{c} -\lambda_{2}-\gamma_{2}-\gamma_{1}-\lambda_{1}p-\mu_{2},\;\;\;i_{2}=s_{2},...,S_{2};j_{2}=i_{2}\\ -\lambda_{2}-\gamma_{1}-\lambda_{1}p-\mu_{2},\;\;\;\;\;\;\;\;i_{2}=S_{2}^{⁎};;j_{2}=i_{2}\\ \gamma_{2},\;\;\;\;\;\;\;\;\;\;\;\;\;i_{2}=s_{2},...,S_{2};j_{2}=S_{2}^{⁎}\\ 0,\;\;\;\;\;\;\;\;\;\;\;\;\;\;\;\;Otherwise \end{array}\right.\;$

$D_{1}^{▿}=D^{▿}+\lambda_{2}I_{l_{1}};Z_{2}^{▿}=Z_{1}^{▿}+\gamma_{1}I_{b};\;\;Z_{4}^{▿}=Z_{3}^{▿}+\gamma_{1}I_{b};\;\;\;\;D_{2}^{▿}=D^{▿}+G^{▿}-C_{1}^{▿};\;\;F^{▿}=\lambda_{1}I_{l_{1}}$;

$G^{▿}=\mu_{2}I_{l_{1}};\;\;L_{2}^{▿}\prime =L_{2}+L_{1}$

$A_{11}^{▿}={\left[\begin{array}{cccccc} D_{2}^{▿} & F^{▿} & 0 & ... & 0 & 0\\ 0 & D^{▿} & F^{▿} & ... & 0 & 0\\ \vdots \\ 0 & 0 & 0 & ... & D^{▿} & F^{▿}\\ 0 & 0 & 0 & ... & 0 & D_{1}^{▿} \end{array}\right]}_{{\overset{ˆ}{d}}_{2}\times {\overset{ˆ}{d}}_{2}}$; $A_{22}^{▿}={\left[\begin{array}{ccccccc} D_{2}^{▿} & F^{▿} & 0 & ... & 0 & 0 & 0\\ G & D^{▿} & F^{▿} & ... & 0 & 0 & 0\\ \vdots \\ 0 & 0 & 0 & ... & G & D^{▿} & F^{▿}\\ 0 & 0 & 0 & ... & 0 & G & D_{1}^{▿} \end{array}\right]}_{\overset{ˆ}{d_{2}}\times \overset{ˆ}{d_{2}}}$

$A_{22}^{{▿}^{\prime }}={\left[\begin{array}{cccccc} D^{▿} & F^{▿} & 0 & ... & 0 & 0\\ G & D^{▿} & F^{▿} & ... & 0 & 0\\ \vdots \\ 0 & 0 & 0 & ... & D^{▿} & F^{▿}\\ 0 & 0 & 0 & ... & G & D_{1}^{▿} \end{array}\right]}_{\overset{ˆ}{d_{2}}\times \overset{ˆ}{d_{2}}}$; $L_{2}^{▿}={\left[\begin{array}{cc} A_{11}^{▿} & A_{12}\\ A_{21} & A_{22}^{{▿}^{\prime }} \end{array}\right]}_{\overset{ˆ}{d_{3}}\times \overset{ˆ}{d_{3}}}$; $L_{2}={\left[\begin{array}{cc} A_{11}^{▿} & A_{12}\\ A_{21} & A_{22}^{▿} \end{array}\right]}_{\overset{ˆ}{d_{3}}\times \overset{ˆ}{d_{3}}}$

Thus the mean sojourn time in the maximum inventory level before attainment of CLT of commodity-1 is given by $E_{S_{1}}=-\gamma_{S_{1}}T_{S_{1}}^{-1}e$, where $\gamma_{S_{1}}$ is a row vector of order $\left(2l_{2}+l_{1}\right)$ and $\gamma_{S_{1}}=\{(0,m,i_{1},i_{2},l),(1,m,i_{1},i_{2},l),...(K,m,i_{1},i_{2},l),(0,0,i_{1},i_{2},0);i_{1}=S_{1}$;

$i_{2}=s_{2},s_{2}+1,...,S_{2},S_{2}^{⁎};\;l=1,2\}$

### 4.6 Mean sojourn time in $S_{2}$ before attainment of common lifetime of commodity-2

We evaluate the mean time in $S_{2}$ before attainment of CLT for commodity-2 in a similar way as the sojourn time in the maximum inventory level before attainment of CLT for commodity-1. Examine the Markov chain $\left\{(N_{4}(t),N_{2}(t),I_{1}(t),I_{2}(t),S(t)\right)$;

t≥0}, over the finite state space. $\left\{(n_{4},n_{2},i_{1},i_{2},r);0\leq n_{4}\leq K\;;i_{2}=S_{2}\;;0\leq n_{2}\leq M\;;s_{1}\leq i_{1}\leq S_{1},S_{1}^{⁎}\;;M\geq 1\;;1\leq r\leq 2\}⋃\{(0,0,i_{1},i_{2},0);s_{1}\leq i_{1}\leq S_{1},S_{1}^{⁎}\;;i_{2}=S_{2}\;;M\geq 1\}⋃{△}_{CLT}^{\prime }.N_{4}(t\right)$ is the number of customers in the pool of finite capacity $K(K>0).{△}_{CLT}^{\prime }$ is an absorbing state which denotes the attainment of common lifetime of commodity-2. All other terms are defined as in section-4.5. The infinitesimal generator of the chain is

$W_{S_{2}}=\left[\begin{array}{cc} \;T_{S_{2}} & T_{S_{2}}^{0}\\ 0 & 0 \end{array}\right]$; $T_{S_{2}}$ is a square matrix of order $\left(2l_{2⁎}+l_{1⁎}\right)$ and $T_{S_{2}}^{0}$ is a matrix of order $(2l_{2⁎}+l_{1⁎})\times 1$. Proceeding as in the case of commodity-1, we get the mean sojourn time in $S_{2}$ before attainment of CLT of commodity-2 is given by $E_{S_{2}}=-\gamma_{S_{2}}T_{S_{2}}^{-1}e$, where $\gamma_{S_{2}}$ is a row vector of order $\left(2l_{2⁎}+l_{1⁎}\right)$ and $\gamma_{S_{2}}=\{(0,m,i_{1},i_{2},l),(1,m,i_{1},i_{2},l),...,(K,m,i_{1},i_{2},l),(0,0,i_{1},i_{2},0)$;

$i_{2}=S_{2};\;0\leq m\leq M,i_{1}=s_{1},s_{1}+1,...,S_{1},S_{1}^{⁎};\;l=1,2\}$. Here $l_{1⁎}=2(S_{1}-s_{1}+1),l_{2⁎}=2(S_{1}-s_{1}+1)(K+1)(M+1)$.

### 4.7 Mean number of revisits to $S_{1}$ before the attainment of common lifetime of commodity-1

To compute the mean number of times the inventory level reaches its maximum level $S_{1}$ for commodity-1 before the attainment of CLT, we consider the Markov chain

$\left\{(N_{5}(t),N_{4}(t),N_{2}(t),I_{1}(t),I_{2}(t),S(t));\;t\geq 0\right\}$ on the state space $\{(n_{5},\;0,\;0,\;i_{1},i_{2}\;0);\;n_{5}\geq 0$;

$i_{1}=s_{1},...,S_{1};\;i_{2}=s_{2},...,S_{2},S_{2}^{⁎}\}⋃\{(n_{5},n_{4},n_{2},i_{1},i_{2}\;1);\;i_{1}=s_{1},...,S_{1};\;i_{2}=s_{2},...,S_{2},S_{2}^{⁎}$;

$0\leq n_{2}\leq M;\;M\geq 1;\;0\leq n_{4}\leq K;\;n_{5}\geq 0\}⋃\{(n_{5},\;n_{4},n_{2}\;i_{1},i_{2}\;2);\;i_{1}=s_{1},...,S_{1};\;i_{2}=s_{2},...,S_{2},S_{2}^{⁎};\;0\leq n_{2}\leq M;\;M\geq 1;\;0\leq n_{4}\leq K;\;n_{5}\geq 0\}⋃\{{△}_{CLT}\}$, where $\left\{{△}_{CLT}\right\}$ is an absorbing state which denotes the attainment of CLT of commodity-1. Here $N_{5}(t)$ denotes the number of revisits to $S_{1}$ up to time t before attainment of CLT for commodity-1. Other terms are as defined as in section-3.1. We consider $N_{5}(t)$ as the level of the process and $N_{4}(t),N_{2}(t),I_{1}(t),I_{2}(t),S(t)$ as the phase. The rate matrix of $\left\{W_{5}(t);t\geq 0\right\}$ is

$W_{5}=\left[\begin{array}{cc} \;\overset{˜}{T} & {\overset{˜}{T}}^{0}\\ 0 & 0 \end{array}\right]\;\;\;;\;\;\overset{˜}{T}=\left[\begin{array}{ccccc} \overset{˜}{R_{2}} & \overset{˜}{R_{1}}\\ & \overset{˜}{R_{2}} & \overset{˜}{R_{1}}\\ & & \overset{˜}{R_{2}} & \overset{˜}{R_{1}}\\ & & & \ddots & \ddots \end{array}\right]$

Let $d_{2}^{\prime \prime }=(S_{1}-s_{1}+1)(S_{2}-s_{2}+2)(M+1)(K+1)$. Here $\overset{˜}{R_{2}},\overset{˜}{R_{1}}$ are square matrices of order $(2d_{2}^{\prime \prime }+{\overset{ˆ}{d}}_{1}).{\overset{˜}{T}}^{0}$ is a column matrix of order $(2d_{2}^{\prime \prime }+{\overset{ˆ}{d}}_{1})\times 1$ and all its elements are $\gamma_{1}$.

$\overset{˜}{R_{2}}=\left[\begin{array}{ccccccc} B_{00}^{⁎} & B_{01}^{⁎} & & & & \\ B_{10} & A_{2}^{⁎} & A_{1}^{⁎}\\ & A_{3}^{⁎} & A_{2}^{⁎} & A_{1}^{⁎}\\ & & \ddots & \ddots & \ddots \\ & & & A_{3}^{⁎} & A_{2}^{⁎} & A_{1}^{⁎}\\ & & & & & A_{3}^{⁎} & A_{2}^{⁎⁎} \end{array}\right]$; $\overset{˜}{R_{1}}=\left[\begin{array}{ccccccc} B_{00}^{⁎⁎} & B_{01}^{⁎⁎} & & & & \\ 0 & 0 & A_{1}^{⁎⁎}\\ & 0 & 0 & A_{1}^{⁎⁎}\\ & & 0 & 0 & A_{1}^{⁎⁎}\\ & & & \ddots & \ddots & \ddots \\ 0 & 0 & 0 & 0 & 0 & 0 & 0 \end{array}\right]$

$B_{00}^{⁎⁎}={\left[\begin{array}{cccccc} 0 & A_{01}^{⁎} & 0 & 0 & .... & 0\\ 0 & 0 & 0 & 0 & .... & 0\\ \vdots \\ 0 & 0 & 0 & 0 & .... & 0 \end{array}\right]}_{\left({\overset{ˆ}{d}}_{3}+{\overset{ˆ}{d}}_{1})\times ({\overset{ˆ}{d}}_{3}+{\overset{ˆ}{d}}_{1}\right)}$;  $E^{\prime \prime }={\left[\begin{array}{ccccc} 0 & 0... & 0 & 0 & 0\\ 0 & E... & 0 & 0 & 0\\ 0 & 0... & E & 0 & 0\\ \vdots \\ 0 & 0... & 0 & E & 0\\ 0 & 0... & 0 & 0 & E \end{array}\right]}_{{\overset{ˆ}{d}}_{1}\times {\overset{ˆ}{d}}_{1}}$

$A_{01}^{⁎}(0,0,i_{1},i_{2},0)\to (1,0,j_{1},j_{2},1)=\left\{ \begin{array}{c} \lambda_{1},\;\;i_{1}=s_{1};s_{2}\leq i_{2}\leq S_{2}^{⁎};j_{1}=S_{1}\\ 0,\;\;\;\;Otherwise \end{array}\right.\;$

$E^{\prime }(0,0,i_{1},i_{2},0)\to (1,0,j_{1},j_{2},1)=\left\{ \begin{array}{c} \lambda_{1}p,\;\;\;i_{1}=s_{1};s_{2}\leq i_{2}\leq S_{2}^{⁎};j_{1}=S_{1}\\ 0,\;\;\;\;\;\;otherwise \end{array}\right.\;$

$C_{01}^{⁎}={\left[\begin{array}{cccccccccc} A_{01}^{⁎} & 0 & 0 & ... & 0 & 0 & 0 & 0 & ... & 0 \end{array}\right]}_{{\overset{ˆ}{d}}_{1}\times {\overset{ˆ}{d}}_{3}};B_{01}^{⁎}={\left[\begin{array}{c} 0\\ A_{1}^{⁎} \end{array}\right]}_{({\overset{ˆ}{d}}_{3}+{\overset{ˆ}{d}}_{1})\times {\overset{ˆ}{d}}_{3}}$

$B_{00}^{⁎}={\left[\begin{array}{cc} C_{00} & C_{01}^{⁎⁎}\\ C_{02} & A_{2}a^{⁎} \end{array}\right]}_{\left({\overset{ˆ}{d}}_{3}+{\overset{ˆ}{d}}_{1})\times ({\overset{ˆ}{d}}_{3}+{\overset{ˆ}{d}}_{1}\right)}\;$; $B_{01}^{⁎⁎}={\left[\begin{array}{c} 0\\ A_{1}^{⁎⁎} \end{array}\right]}_{({\overset{ˆ}{d}}_{3}+{\overset{ˆ}{d}}_{1})\times {\overset{ˆ}{d}}_{3}}$

$C_{01}^{⁎⁎}={\left[\begin{array}{cccccccccc} A_{01}^{⁎⁎} & 0 & 0 & ... & 0 & 0 & 0 & 0 & ... & 0 \end{array}\right]}_{{\overset{ˆ}{d}}_{1}\times {\overset{ˆ}{d}}_{3}};\;\;\;A_{2}^{⁎⁎}=A_{2}^{⁎}+A_{1}^{⁎}$

$A_{01}^{⁎⁎}(0,0,0,i_{1},i_{2},0)\to (1,0,0,j_{1},j_{2},1)=\left\{ \begin{array}{c} \lambda_{1},\;\;\;\;i_{1}=s_{1}+1,...,S_{1};\;s_{2}\leq i_{2}\leq S_{2}^{⁎}\;;\;j_{1}=i_{1}-1\\ 0,\;\;\;\;\;\;\;\;otherwise \end{array}\right.\;$

$A_{1}^{⁎}$ is a diagonal matrix with diagonal blocks $\left(E^{\prime \prime },E,E,...,E,E^{\prime \prime \;},E,E,...,E\right)$ and $A_{1}^{⁎⁎}$ is a diagonal matrix with diagonal blocks $\left(E^{\prime },0,0,...,0,E^{\prime },0,0,...,0\right)$. Entries of $A_{2}^{⁎}$ are similar to $A_{2}$, entries of $A_{2a}^{⁎}$ are similar to $A_{2}^{\prime }$ and entries of $A_{3}^{⁎}$ are similar to $A_{3}$, only their dimension reduces to ${\overset{ˆ}{d}}_{3}\times {\overset{ˆ}{d}}_{3}$. Proceeding as in [32] and define $P_{k}$ as the probability that absorption occurs with precisely *k* revisits, then $P_{k}=\alpha_{K}{\left(-{\overset{˜}{R_{2}}}^{-1}\overset{˜}{R_{1}}\right)}^{k}(-{\overset{˜}{R_{2}}}^{-1}\overset{˜}{R_{1}}),k\geq 0$ with $\alpha_{K}=({\text{x}}_{0},{\text{x}}_{1},...,{\text{x}}_{K})$ is a row vector of order $\left(K+1)(2d_{2}^{\prime \prime }+{\overset{ˆ}{d}}_{1}\right)$. Hence the mean number of revisits to $S_{1}$ before the attainment of CLT is $\sum_{k=0}^{\infty }\;kP_{k}$.

### 4.8 Mean number of revisits to $S_{2}$ before the attainment of common lifetime of commodity-2

We compute the mean number of revisits to $S_{2}$ before the attainment of CLT of commodity-2 in a similar way as the mean number of revisits to $S_{1}$ before the attainment of CLT of commodity-1. Consider the Markov chain $\left\{N_{6}(t),N_{4}(t),N_{2}(t),I_{1}(t),I_{2}(t),S(t);t\geq 0\right\}$ over the state space, $\left\{(n_{6},\;0,\;0,\;i_{1},i_{2}\;,0);n_{6}\geq 0\;,i_{1}=s_{1},...,S_{1},S_{1}^{⁎}\;;i_{2}=s_{2},...,S_{2}\}\cup \{(n_{6},\;n_{4},n_{2},i_{1},i_{2},1);i_{1}=s_{1},...,S_{1},S_{1}^{⁎}\;;\;i_{2}=s_{2},...,S_{2}\;;\;0\leq n_{2}\leq M\;;\;M\geq 1;\;0\leq n_{4}\leq K;\;n_{6}\geq 0\}\cup \{(n_{6},n_{4},n_{2},i_{1},i_{2},2)\;;\;i_{1}=s_{1},...,S_{1}\;,S_{1}^{⁎}\;;\;i_{2}=s_{2},...,S_{2}\;;\;0\leq n_{2}\leq M\;;\;M\geq 1\;;0\leq n_{4}\leq K\;;n_{6}\geq 0\}\cup \{{△}_{CLT}\right\}$, where $\left\{{△}_{CLT}\right\}$ is an absorbing state which represent the attainment of CLT of commodity-2. The symbol $N_{6}(t)$ denotes the number of revisits to $S_{2}$ up to time *t* before attainment of CLT for commodity-2. Other terms are same as in section 4.7. The rate matrix of the chain is

$W_{R_{2}}=\left[\begin{array}{cc} \overset{˜}{\overset{ˆ}{T}} & {\overset{˜}{\overset{ˆ}{T}}}^{0}\\ 0 & 0 \end{array}\right]$;  $\overset{˜}{\overset{ˆ}{T}}=\left[\begin{array}{ccccc} \overset{˜}{\overset{ˆ}{R_{2}}} & \overset{˜}{\overset{ˆ}{R_{1}}}\\ & \overset{˜}{\overset{ˆ}{R_{2}}} & \overset{˜}{\overset{ˆ}{R_{1}}}\\ & & \overset{˜}{\overset{ˆ}{R_{2}}} & \overset{˜}{\overset{ˆ}{R_{1}}}\\ & & & \ddots & \ddots \end{array}\right]$

and the matrices $\overset{˜}{\overset{ˆ}{R_{2}}},\overset{˜}{\overset{ˆ}{R_{1}}}$ etc can be defined as in the case of commodity-1. Proceeding as in the case of commodity-1, and define $P_{k}^{▵}$ as the probability that absorption occurs with precisely k revisits, then $P_{k}^{▵}=\alpha_{K}{\left(-{\overset{˜}{\overset{ˆ}{R}}}_{2}^{-1}\overset{˜}{\overset{ˆ}{R_{1}}}\right)}^{k}(-{\overset{˜}{\overset{ˆ}{R_{2}}}}^{-1}\overset{˜}{\overset{ˆ}{R_{1}}}),k\geq 0$ with $\alpha_{K}=({\text{x}}_{0},{\text{x}}_{1},...,{\text{x}}_{K})$ is a row vector of order $\left(K+1)(2{\overset{ˆ}{d}}_{2}^{\prime \prime }+{\overset{ˆ}{d}}_{1}^{\prime }\right)$. Therefore the mean number of revisits to $S_{2}$ before the attainment of CLT is $\sum_{k=0}^{\infty }\;kp_{k}^{▵}$.

## 5 Numerical illustration

In this section we provide numerical illustration of the system performance measures with various values of the underlying parameters.

**Influence of** $\lambda_{1}$ **on various performance measures**

We study the influence of $\lambda_{1}$ for fixed values of $p=1/6,M=5,(s_{1},S_{1})=(s_{2},S_{2})=(3,5),\lambda_{2}=19,\mu_{1}=12.7,\mu_{2}=14,\gamma_{1}=10,\gamma_{2}=15$, on various measures of performance and are given in Table 1. As $\lambda_{1}$ increases, we observe an increase in expected values of number of type-1 (non priority) customers, average waiting time of type-1 customers and average loss rate of type-1 customers.

**Table 1:** Influence of ***λ***_**1**_ on various performance measures.

*λ*_1_	*EN*_1_	*Ew*_1_	*EL*_1_
5.3	0.2067	0.2625	0.0115
5.4	0.3204	0.4779	0.0207
5.5	0.4643	0.6769	0.0298
5.6	0.6065	0.8582	0.0386
5.7	0.7462	1.0295	0.0471

**Influence of** $\mu_{1}$ **on various performance measures**

Here we study the influence of $\mu_{1}$ for fixed values of $p=1/6,M=5,(s_{1},S_{1})=(s_{2},S_{2})=(3,5),\lambda_{1}=5.2,\lambda_{2}=19,\mu_{2}=14,\gamma_{1}=10,\gamma_{2}=15$, on various performance measures and are given in Table 2. As $\mu_{1}$ increases, a decrease in expected values of number of type-1(non priority) customers, average waiting time of type-1 customers and average loss rate of type-1 customers. Also as $\mu_{1}$ increases, the server busy probability increases and probability of server idle decreases.

**Table 2:** Influence of ***μ***_**1**_ on various performance measures.

*μ*_1_	*EN*_1_	*Ew*_1_	*EL*_1_	P(busy)	P(idle)
12.0	0.4979	0.6864	0.0298	0.9966	0.0034
12.1	0.4249	0.5967	0.0258	0.9968	0.0032
12.2	0.3540	0.5073	0.0217	0.9971	0.0029
12.3	0.2865	0.4151	0.0177	0.9973	0.0027
12.4	0.2292	0.3222	0.0137	0.9975	0.0025

**Influence of** $\lambda_{2}$ **on various performance measures**

Here we study the influence of $\lambda_{2}$ for fixed values of $p=1/6,M=5,(s_{1},S_{1})=(3,5),(s_{2},S_{2})=(7,9),\lambda_{1}=5.6,\mu_{1}=13.1,\mu_{2}=13.8,\gamma_{1}=10,\gamma_{2}=15$ on various performance measures and are given in Table 3. As $\lambda_{2}$ increases, we notice that an increase in expected number of type-1 and type-2 customers, expected loss rate of type-1 and type-2 customers and expected waiting time of type-1 and type-2 customers.

**Table 3:** Influence of ***λ***_**2**_ on various performance measures.

*λ*_2_	*EN*_1_	*EN*_2_	*Ew*_1_	*Ew*_2_	*EL*_1_	*EL*_2_
18.9	0.5640	0.0810	0.5264	0.0031	0.0378	0.1337
19.0	0.6879	0.0982	0.6314	0.0032	0.0461	0.1639
19.1	0.8075	0.1148	0.7300	0.0033	0.0542	0.1936
19.2	0.9242	0.1308	0.8228	0.0034	0.0621	0.2228
19.3	1.0376	0.1462	0.9102	0.0035	0.0697	0.2516

**Influence of** $\mu_{2}$ **on various performance measures**

Here we study the influence of $\mu_{2}$ for fixed values of $p=1/6,M=5,(s_{1},S_{1})=(3,5),(s_{2},S_{2})=(7,9),\lambda_{1}=5.6,\lambda_{2}=18.8,\mu_{1}=12.8,\gamma_{1}=10,\gamma_{2}=15$ on various performance measures and are given in Table 4. As $\mu_{2}$ increases, we observe a decrease in the expected number of type-1 and type-2 customers, expected loss rate of type-1 and type-2 customers and average waiting time of type-1 and type-2 customers. Also as $\mu_{2}$ increases, the server busy probability increases and probability of server idle decreases.

**Table 4:** Influence of ***μ***_**2**_ on various performance measures.

*μ*_2_	*EN*_1_	*EN*_2_	*Ew*_1_	*Ew*_2_	*EL*_1_	*EL*_2_	P(busy)	P(idle)
13.4	1.2634	0.1715	1.1120	0.0036	0.0821	0.2869	0.9970	0.0030
13.5	1.1138	0.1518	0.9890	0.0035	0.0723	0.2524	0.9973	0.0027
13.6	0.9576	0.1311	0.8584	0.0034	0.0620	0.2166	0.9976	0.0024
13.7	0.7590	0.1093	0.7198	0.0033	0.0514	0.1796	0.9979	0.0021
13.8	0.6261	0.0865	0.5730	0.0031	0.0404	0.1412	0.9983	0.0017

**Influence of** $\gamma_{1}$ **on various performance measures**

Here we study the influence of $\gamma_{1}$ for fixed values of $p=1/6,M=3,(s_{1},S_{1})=(1,6),(s_{2},S_{2})=(3,8),\lambda_{1}=28,\lambda_{2}=29,\mu_{1}=42,\mu_{2}=44,\gamma_{2}=10$ on the system performance measures and are given in Table 5. As $\gamma_{1}$ increases, we observe an increase in expected number of reorders of commodity-1 $\left(\sigma_{1}\right)$ due to CLT realization in a cycle, total expected reorder rate of commodity-1 $\left(E_{R_{1}}\right)$ and a decrease in mean cycle length of commodity-1 $\left(\eta_{1}\right)$.

**Table 5:** Influence of ***γ***_**1**_ on various performance measures.

*γ*_1_	10	10.1	10.2	10.3	10.4	10.5	10.6	10.7
*ER*_1_	12.6720	12.7820	12.9121	13.0446	13.1646	13.3011	13.4401	13.5801
*η*_1_	0.5522	0.5521	0.5519	0.5516	0.5513	0.5510	0.5507	0.5504
*σ*_1_	7	7.0928	7.1828	7.2928	7.4028	7.5128	7.6328	7.7178

**Influence of** $\gamma_{2}$ **on various performance measures**

Here we study the influence of $\gamma_{2}$ for fixed values of  on various performance measures and are given in Table 6. As $\gamma_{2}$ increases, we observe an increase in expected number of reorders of commodity-2 $\left(\sigma_{2}\right)$ due to CLT realization in a cycle, total expected reorder rate of commodity-2 $\left(E_{R_{2}}\right)$ and a decrease in mean cycle length of commodity-2 $\left(\eta_{2}\right)$.

**Table 6:** Influence of ***γ***_**2**_ on various performance measures.

*γ*_2_	15	15.1	15.2	15.3	15.4	15.5	15.6	15.7
*ER*_2_	1.4900	1.5020	1.5145	1.5275	1.5409	1.5541	1.5677	1.5811
*η*_2_	0.3261	0.3261	0.3260	0.3260	0.3259	0.3258	0.3258	0.3258
*σ*_2_	1	1.0002	1.0004	1.0004	1.0005	1.0006	1.0006	1.0007

### 5.1 Cost optimization

Based on the performance measures obtained, we build a cost function for checking the optimality of the control parameters $s_{i}$ and $S_{i},i=1,2$. We consider the following costs to compute the total expected cost per unit of time.

$Ch_{i}$ - Cost of holding inventory per unit of time for commodity-i,i=1,2

$Cw_{i}$ - Waiting cost in the system per unit time of type-*i* customer, i=1,2

$Cr_{i}$ - Purchase price per unit for commodity-i,i=1,2

$Cl_{i}$ - Cost due to non-priority and priority customer loss per unit of time, i=1,2

$Ch_{i}$ - Holding cost of non-priority and priority customer per unit of time in the pool and in the buffer respectively for i=3,4. Then total expected cost is defined as

$T(s_{1},s_{2};S_{1},S_{2})=Ch_{1}.EI_{1}+Ch_{2}.EI_{2}+Cw_{1}.Ew_{1}+Cw_{2}.Ew_{2}+Cr_{1}.E_{R_{1}}+Cr_{2}.E_{R_{2}}+Cl_{1}.EL_{1}+Cl_{2}.EL_{2}+Ch_{3}.EN_{1}+Ch_{4}.EN_{2}$.

**Figure 1:**
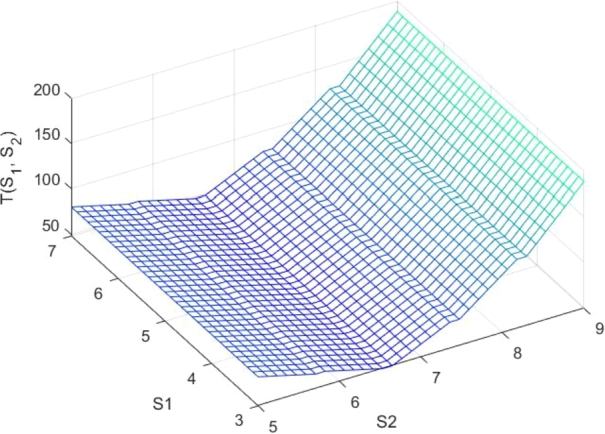
Total expected cost per unit time for various values of *S*_1_ and *S*_2_.

Due to the intricacy of the cost function it is difficult to obtain the global optimum values of $s_{i}$ and $S_{i}$. We look the convexity of the cost function numerically. For the given fixed values of the parameters and fixed values of $s_{1}$ and $s_{2}$, we computed the total expected cost per unit time for different values of $S_{1}$ and $S_{2}$ and are given in Table 7 (Fig. 1)

**Table 7:** Total expected cost per unit time for various values of *S*_1_ and *S*_2_.

(*s*_1_,*S*_1_)	(1,3)	(1,4)	(1,5)	(1,6)	(1,7)	(1,8)	(1,9)	(1,10)
(*s*_2_,*S*_2_)	(3,5)	(3,6)	(3,7)	(3,8)	(3,9)	(3,10)	(3,11)	(3,12)
*T*(*s*_1_,*s*_2_;*S*_1_,*S*_2_)	80.54	64.52	48.40	77.60	124.92	188.10	258.53	338.09

Fix values: $\mu_{1}=14;\mu_{2}=15;\gamma_{1}=15;\gamma_{2}=2;p=1/4;\lambda_{1}=8;\lambda_{2}=9;M=3$;

Fixed costs: $Cl_{1}=5;Cl_{2}=10;Cr_{1}=1;Cr_{2}=2;Cw_{1}=1;Cw_{2}=5;Ch_{1}=6$;

$Ch_{2}=8;Ch_{3}=2;Ch_{4}=6$;

The numerical values show that $T(s_{1},s_{2};S_{1},S_{2})$ is minimum when $S_{1}=5$ and $S_{2}=7$ for fixed value of $s_{1}=1$ and $s_{2}=3$.

**Figure 2:**
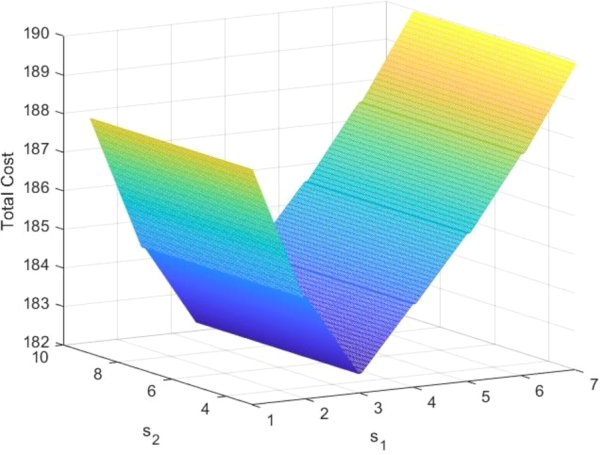
Total expected cost per unit time for various values of *s*_1_ and *s*_2_.

For the given fixed values of the parameters and fixed values of $S_{1}$ and $S_{2}$, we computed the total expected cost per unit time for different values of $s_{1}$ and $s_{2}$ and are given in Table 8 (Fig. 2)

**Table 8:** Total expected cost per unit time for various values of *s*_1_ and *s*_2_.

(*s*_1_,*S*_1_)	(1,8)	(2,8)	(3,8)	(4,8)	(5,8)	(6,8)	(7,8)
(*s*_2_,*S*_2_)	(3,10)	(4,10)	(5,10)	(6,10)	(7,10)	(8,10)	(9,10)
*T*(*s*_1_,*s*_2_;*S*_1_,*S*_2_)	188.10	184.62	182.52	184.17	185.85	187.76	189.94

Fix values: $\mu_{1}=14;\mu_{2}=15;\gamma_{1}=15;\gamma_{2}=2;p=1/4;\lambda_{1}=8;\lambda_{2}=9;M=3$;

Fixed costs: $Cl_{1}=5;Cl_{2}=10;Cr_{1}=1;Cr_{2}=2;Cw_{1}=1;Cw_{2}=5;Ch_{1}=6$;

$Ch_{2}=8;Ch_{3}=2;Ch_{4}=6$;

The numerical values show that $T(s_{1},s_{2})$ is minimum when $s_{1}=3$ and $s_{2}=5$ for fixed value of $S_{1}=8$ and $S_{2}=10$.

**Influence of common lifetime parameters on the cost function**

Here, we study the effect of common lifetime parameters $\gamma_{i},i=1,2$ on total expected cost. From Table 9, Table 10, we get that as the rate of common lifetime parameters $\gamma_{1}$ and $\gamma_{2}$ increases, the total expected cost per unit time also increases

**Table 9:** Influence of common lifetime parameter ***γ***_**1**_ on the cost function.

*γ*_1_	10	15	20	25	30	35	40	45
*T*(*s*_1_,*s*_2_;*S*_1_,*S*_2_)	56.371	57.100	57.653	58.148	58.622	59.088	59.550	60.010

Fix: $p=1/4;(s_{1},S_{1})=(1,5);(s_{2},S_{2})=(3,7);M=3;\mu_{1}=14;\mu_{2}=15;\lambda_{1}=8;\gamma_{2}=2;\lambda_{2}=13$.

**Table 10:** Influence of common lifetime parameter ***γ***_**2**_ on the cost function.

*γ*_2_	10	13	16	19	22	25	28	31
*T*(*s*_1_,*s*_2_;*S*_1_,*S*_2_)	51.067	52.661	54.252	55.841	57.431	59.023	60.616	62.211

Fix: $p=1/4;(s_{1},S_{1})=(1,5);(s_{2},S_{2})=(3,7);M=3;\mu_{1}=14;\mu_{2}=15;\lambda_{1}=8;\gamma_{1}=10;\lambda_{2}=13$.

## 6 Conclusion

In this paper, we have studied a two commodity queueing inventory system at a service facility with a common lifetime for each of the commodities, zero lead time, and positive service time, and two classes of customers. Also, there is a finite capacity buffer for priority customers and an infinite pool for non-priority customers. The matrix geometric method is used for finding the steady state probability vector. We have studied various system characteristics such as the expected number of customers, the expected loss rate of customers, the expected waiting time of customers, expected inventory levels, expected reorder rates for commodities, mean sojourn time at the maximum inventory level before attainment of the common lifetime, mean number of revisits to the maximum inventory level before attainment of the common lifetime, and analyzed the mean cycle time for both commodities. We have presented numerical illustrations to show the effect of various parameters on performance measures. A cost function is constructed and analyzed numerically to find the optimum values of the control parameters $s_{i}$ and $S_{i},i=1,2$. Also, a sensitivity analysis of the common lifetime parameters is done to show the effect on the cost function. The present work can be extended by adding positive random lead times for inventory replenishment.

## CRediT authorship contribution statement

**S. Dissa:** Writing – review & editing, Writing – original draft, Visualization, Validation, Software, Methodology, Investigation, Formal analysis, Data curation. **P.V. Ushakumari:** Writing – review & editing, Writing – original draft, Visualization, Validation, Supervision, Software, Methodology, Investigation, Formal analysis, Data curation, Conceptualization.

## Declaration of Competing Interest

The authors declare no conflict of interest.

## Data Availability

No data was used for the research described in the article.
